# Switch from antagonist to agonist of the androgen receptor blocker bicalutamide is associated with prostate tumour progression in a new model system

**DOI:** 10.1038/sj.bjc.6690684

**Published:** 1999-09

**Authors:** Z Culig, J Hoffmann, M Erdel, I E Eder, A Hobisch, A Hittmair, G Bartsch, G Utermann, M R Schneider, K Parczyk, H Klocker

**Affiliations:** Departments of Urology, Medical Biology and Human Genetics, Pathology, University of Innsbruck, Anichstraße 35, Innsbruck, A-6020, Austria; Research Laboratories of Schering AG, Berlin, D-13342, Germany

**Keywords:** prostate cancer, androgen ablation, LNCaP cells, androgen receptor, bicalutamide, tumour progression

## Abstract

Advanced prostate cancer is treated by androgen ablation and/or androgen receptor (AR) antagonists. In order to investigate the mechanisms relevant to the development of therapy-resistant tumours, we established a new tumour model which closely resembles the situation in patients who receive androgen ablation therapy. Androgen-sensitive LNCaP cells were kept in androgen-depleted medium for 87 passages. The new LNCaP cell subline established in this manner, LNCaP-abl, displayed a hypersensitive biphasic proliferative response to androgen until passage 75. Maximal proliferation of LNCaP-abl cells was achieved at 0.001 nM of the synthetic androgen methyltrienolone (R1881), whereas 0.01 nM of this compound induced the same effect in parental cells. At later passages (> 75), androgen exerted an inhibitory effect on growth of LNCaP-abl cells. The non-steroidal anti-androgen bicalutamide stimulated proliferation of LNCaP-abl cells. AR protein expression in LNCaP-abl cells increased approximately fourfold. The basal AR transcriptional activity was 30-fold higher in LNCaP-abl than in LNCaP cells. R1881 stimulated reporter gene activity in LNCaP-abl cells even at 0.01 nM, whereas 0.1 nM of R1881 was needed for induction of the same level of reporter gene activity in LNCaP cells. Bicalutamide that acts as a pure antagonist in parental LNCaP cells showed agonistic effects on AR transactivation activity in LNCaP-abl cells and was not able to block the effects of androgen in these cells. The non-steroidal AR blocker hydroxyflutamide exerted stimulatory effects on AR activity in both LNCaP and LNCaP-abl cells; however, the induction of reporter gene activity by hydroxyflutamide was 2.4- to 4-fold higher in the LNCaP-abl subline. The changes in AR activity were associated neither with a new alteration in AR cDNA sequence nor with amplification of the AR gene. Growth of LNCaP-abl xenografts in nude mice was stimulated by bicalutamide and repressed by testosterone. In conclusion, our results show for the first time that the non-steroidal anti-androgen bicalutamide acquires agonistic properties during long-term androgen ablation. These findings may have repercussions on the natural course of prostate cancer with androgen deprivation and on strategies of therapeutic intervention. © 1999 Cancer Research Campaign


*British Journal of Cancer* (1999) **81**(2), 242–251
© 1999 Cancer Research Campaign
Article no. bjoc.1999.0684

### Switch from antagonist to agonist of the androgen receptor blocker bicalutamide is associated with prostate tumour progression in a new model system

**Z Culig[1,*], J Hoffmann[4,*], M Erdel[2], IE Eder[1], A Hobisch[1], A Hittmair[3], G Bartsch[1], G Utermann[2], MR Schneider[4], K Parczyk[4] and H Klocker[1]**

Departments of [1]Urology, [2]Medical Biology and Human Genetics and [3]Pathology, University of Innsbruck, Anichstraße 35, A-6020 Innsbruck, Austria; [4]Research Laboratories of Schering AG, D-13342 Berlin, Germany

**Summary** Advanced prostate cancer is treated by androgen ablation and/or androgen receptor (AR) antagonists. In order to investigate the mechanisms relevant to the development of therapy-resistant tumours, we established a new tumour model which closely resembles the situation in patients who receive androgen ablation therapy. Androgen-sensitive LNCaP cells were kept in androgen-depleted medium for 87 passages. The new LNCaP cell subline established in this manner, LNCaP-abl, displayed a hypersensitive biphasic proliferative response to androgen until passage 75. Maximal proliferation of LNCaP-abl cells was achieved at 0.001 nM of the synthetic androgen methyltrienolone (R1881), whereas 0.01 nM of this compound induced the same effect in parental cells. At later passages (> 75), androgen exerted an inhibitory effect on growth of LNCaP-abl cells. The non-steroidal anti-androgen bicalutamide stimulated proliferation of LNCaP-abl cells. AR protein expression in LNCaP-abl cells increased approximately fourfold. The basal AR transcriptional activity was 30-fold higher in LNCaP-abl than in LNCaP cells. R1881 stimulated reporter gene activity in LNCaP-abl cells even at 0.01 nM, whereas 0.1 nM of R1881 was needed for induction of the same level of reporter gene activity in LNCaP cells. Bicalutamide that acts as a pure antagonist in parental LNCaP cells showed agonistic effects on AR transactivation activity in LNCaP-abl cells and was not able to block the effects of androgen in these cells. The non-steroidal AR blocker hydroxyflutamide exerted stimulatory effects on AR activity in both LNCaP and LNCaP-abl cells; however, the induction of reporter gene activity by hydroxyflutamide was 2.4- to 4-fold higher in the LNCaP-abl subline. The changes in AR activity were associated neither with a new alteration in AR cDNA sequence nor with amplification of the AR gene. Growth of LNCaP-abl xenografts in nude mice was stimulated by bicalutamide and repressed by testosterone. In conclusion, our results show for the first time that the non-steroidal anti-androgen bicalutamide acquires agonistic properties during long-term androgen ablation. These findings may have repercussions on the natural course of prostate cancer with androgen deprivation and on strategies of therapeutic intervention.

© 1999 Cancer Research Campaign

**Keywords:** prostate cancer; androgen ablation; LNCaP cells; androgen receptor; bicalutamide; tumour progression

Although prostate cancer remains the most common male malignant tumour in the Western world, there is still a lack of a sufficient number of models for studying its development and progression (Dijkman and Debruyne, 1996). The three human cell lines, LNCaP, PC-3 and DU-145, all derived from metastatic lesions from prostate cancer, are widely used (Stone et al, 1978; Kaighn et al, 1979; Horoszewicz et al, 1983). Two of these three cell lines are not representative of androgen receptor (AR) expression in prostate cancer in vivo (Hobisch et al, 1995). PC-3 and DU-145 cells express very little or no AR, respectively, whereas advanced prostate tumours and metastatic lesions from patients who failed androgen ablation therapy were found to be altogether AR-positive (Tilley et al, 1990; van der Kwast et al, 1991; Hobisch et al, 1995; Taplin et al, 1995). On the other hand, LNCaP cells, obtained from a lymph node metastasis of a patient who did not respond to endocrine therapy, contain high levels of AR mRNA and protein and are androgen-sensitive. The LNCaP AR, which has a point mutation in the hormone-binding domain, binds various steroids (oestradiol, progesterone) and non-steroidal anti-androgens (hydroxyflutamide, nilutamide) with higher affinity than the wild-type AR (Veldscholte et al, 1990). These substances, in addition to androgens, induce transcriptional activity of the LNCaP AR. LNCaP cells grow in response to androgenic, oestrogenic and progestagenic steroids, hydroxyflutamide and nilutamide (Schuurmans et al, 1988; Wilding et al, 1989). Conversely, agonistic effects on LNCaP proliferation and AR activity were not observed in the presence of another non-steroidal anti-androgen, bicalutamide (Casodex) (Veldscholte et al, 1992). The proliferation of LNCaP cells is regulated by androgens in a biphasic manner. It is enhanced by low concentrations of dihydrotestosterone (DHT), whereas higher androgen doses induce a progressive decline in cellular proliferation. They induce expression of prostate-specific genes, such as the prostate-specific antigen (PSA) gene (Lee et al, 1995).

Androgen ablation therapy for advanced prostatic carcinoma intends to block AR activation and function. Serum levels of testicular androgens are thus lowered either by orchiectomy or by analogues of luteinizing hormone-releasing hormone. These compounds are applied together with an AR antagonist in order to achieve a complete androgen blockade. The response to this

*Received 1 December 1998*
*Revised 13 April 1999*
*Accepted 22 April 1999*

*Correspondence to:* H Klocker

*Joint first authors of the manuscript.

therapy is time-limited. Almost all tumours become refractory after some time. Surprisingly, clinical benefit has been observed following the withdrawal of anti-androgens in a subset of prostate cancer patients with therapy-resistant disease (Scher and Kolvenbag, 1997). This anti-androgen withdrawal phenomenon may be in part attributed to mutant ARs detected in prostatic carcinomas but alternative mechanisms may also be considered. These facts indicate that there may be clinically relevant changes in AR expression and function during long-term androgen ablation. We report here on characterization of a new LNCaP cell subline, LNCaP-abl, which was established after 10 months propagation in steroid-depleted medium. This characterization encompasses the evaluation of in vitro and in vivo tumour growth, the expression and activity of the AR and cytogenetic changes. Our study demonstrates a hyperresponsiveness of the androgen-signalling pathway in long-term androgen-deprived LNCaP cells.

### MATERIALS AND METHODS

### Materials

RPMI-1640 medium was purchased from HyClone (Logan, UT, USA). Fetal calf serum (FCS) and penicillin–streptomycin were from Biological Industries (Kibutz Beth Haemek, Israel). Cell culture plasticware was from Falcon (Lincoln, NE, USA), Costar (Cambridge, MA, USA) and Sarstedt (Nümbrecht, Germany). Non-radioactive and $^3$H-labelled synthetic androgen methyltrienolone (R1881, specific activity 86 Ci mmol$^{-1}$) and acetyl-coenzyme A (specific activity 57 mCi mmol$^{-1}$) were purchased from New England Nuclear (Dreieichenhain, Germany). Hydroxyflutamide was from Essex Pharma (Munich, Germany) and bicalutamide from Schering AG (Berlin, Germany). Chloramphenicol and unlabelled acetyl-coenzyme A were supplied by Sigma (Deisenhofen, Germany). The scintillation liquids Optiphase and Optiscint ‘Hisafe’ were from Pharmacia (Uppsala, Sweden). A commercial methylthiotetrazole (MTT) assay was purchased from Biomedica (Vienna, Austria). A luciferase assay kit was purchased from Bio Orbit (Turku, Finland). Electroporation apparatus and cuvettes were from Eurogentec (Brussels, Belgium). The softwares for karyotyping and comparative genomic hybridization (CGH) were from Perceptive Scientific International (Chester, UK). Biotin- and digoxigenin-labelled dUTPs were from Boehringer (Mannheim, Germany). The Leitz Aristoplan epifluorescence microscope (Leica, Vienna, Austria) was equipped with a cooled integrating black and white charge-coupled device (CCD) Image Point camera (Photometrics, Tucson, AZ, USA). A triple band-pass filter set was from Chroma Technology (Brattleboro, VA, USA). The Polyvar 2 microscope from Reichert (Vienna, Austria) was equipped with a video camera (Hamamatsu Photonics, Hamamatsu, Japan).

### Cell culture

The LNCaP cell line was obtained at passage 21 from the American Type Culture Collection (Bethesda, MD, USA). The cells were routinely cultured in RPMI medium supplemented with 10% FCS and antibiotics at 37°C in an atmosphere of 5% carbon dioxide in air. Medium was changed every 3–4 days and the cells were passaged by trypsinization once a week with ratio 1:3. Steroid removal was performed in two steps; initially, FCS was incubated with dextran-coated charcoal at 4°C for 5 h. The charcoal mixture was centrifuged at 3600 rpm for 20 min. Then the supernatant was incubated with a fresh portion of dextran-coated charcoal overnight, centrifuged, filtered through a 0.2 μm sterile filter and stored at –20°C until use. Beginning with passage 25, we have cultured LNCaP cells in 10% charcoal-stripped (CS) FCS and antibiotics with medium change every 3–4 days and passage once a week. The levels of testosterone, oestradiol and progesterone in CS FCS were determined by commercial radioimmunoassays (RIAs). The testosterone RIA (sensitivity 0.14 nM) was purchased from Diagnostic Products Corporation (Los Angeles, CA, USA), RIA for determination of oestradiol (sensitivity 0.05 nM) was from Serono (Roma, Italy) and the progesterone RIA (sensitivity 0.3 nM) was from Orion Diagnostica (Espoo, Finland). Testosterone, oestradiol and progesterone were not detected in CS FCS by these RIAs. The cells derived after 41 passages in steroid-free medium were named LNCaP-abl. Several passages of these cells were characterized. Low passages of LNCaP cells (25–36), which were not exposed to steroid deprivation, served as controls.

### Assessment of in vitro proliferation of LNCaP cells

Cell proliferation in the absence of any supplement was evaluated by counting trypsinized cells with a haemocytometer. The cells were counted after (a) culture of LNCaP cells in FCS and LNCaP-abl cells in CS FCS, (b) culture of both cell lines in FCS and (c) culture of both cell lines in CS FCC. Responsiveness of cell proliferation to increasing concentrations of androgen and anti-androgens was evaluated by means of the MTT (3-(4,5-dimethylthiazol-2-yl)-2,5-diphenyltetrazolium bromide) assay. It was previously found that MTT converting activity adequately reflects cell number in androgen-treated LNCaP cells (Romijn et al, 1988). LNCaP cells were seeded into 24-well plates at a density of $2 \times 10^4$ per well. They were grown in medium containing 10% CS FCS for 24 h. After this period, the concentration of CS FCS was reduced to 5% and either androgen or one of the non-steroidal anti-androgens (hydroxyflutamide, bicalutamide) was added. Proliferative response was assessed after 72 h. The MTT assay was performed as recently described by Cronauer et al (1996).

### Ligand binding assay

About $3 \times 10^6$ cells were seeded into 75-cm$^2$ flasks and incubated for 48 h. Then they were taken to determine specific binding of radioactively labelled methyltrienolone. The assay procedure was essentially the same as that described previously (Culig et al, 1993*a*). In addition to determination of androgen binding, competition studies with bicalutamide were carried out and relative binding affinity was calculated.

### AR transcriptional activity

Activity of the AR was assessed in transactivation assays. The reporter chloramphenicol acetyltransferase (CAT) gene, driven by a promoter consisting of two androgen-response elements in front of a TATA box (Culig et al, 1994), was introduced together with the plasmid pGL$_3$-control, which contains a luciferase gene driven by the SV-40 promoter (Promega, Mannheim, Germany) into LNCaP and LNCaP-abl cells by means of electroporation. Prior to electroporation, the cells were grown in 75-cm$^2$ flasks for 24 h. They were washed in phosphate-buffered saline (PBS) and trypsinized. Then $3.6 \times 10^6$ cells were resuspended in 400 μl of

© 1999 Cancer Research Campaign

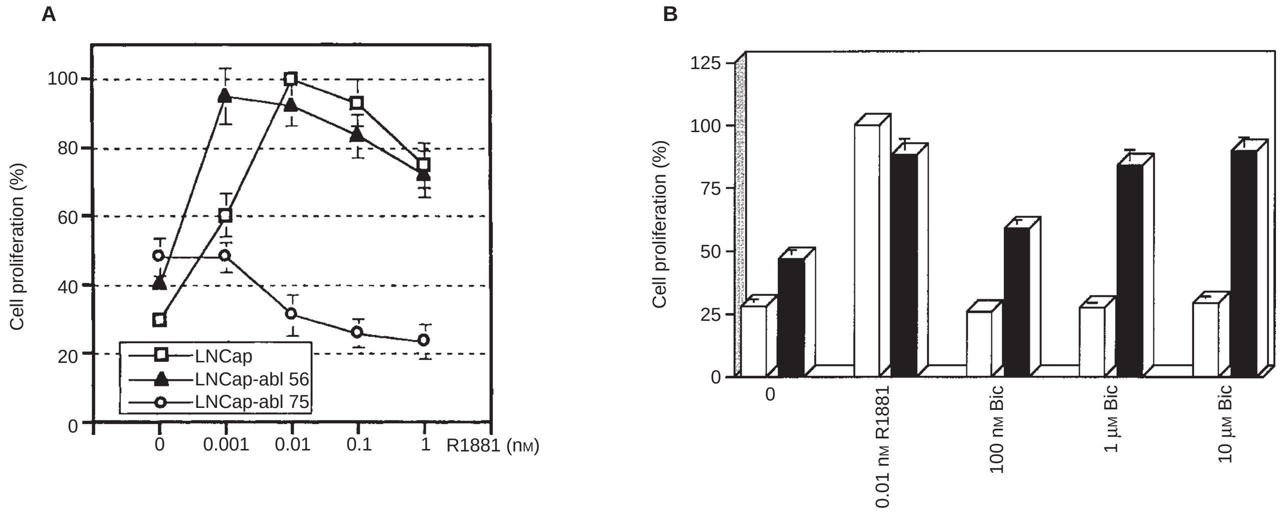

**Figure 1** Proliferative response of LNCaP and LNCaP-abl cells following androgen or bicalutamide treatment. Two × $10^4$ cells were seeded into 24-well plates and grown in the presence of indicated concentrations of R1881 or bicalutamide for 72 h. Proliferation was assessed by means of the MTT assay. Results are expressed in relation to LNCaP cells stimulated by 0.01 nM of R1881 (=100%). (**A**) The effects of R1881 on LNCaP and LNCaP-abl cells (passages 56 and 75). Points, mean values of three experiments; bars, s.d. (**B**) The effects of bicalutamide (Bic) on LNCaP (white bar) and LNCaP-abl cells (passage 68) (black bar)

PBS containing 10% CS FCS and mixed with 60 μg of the reporter plasmid and 40 μg of the control plasmid. This suspension was transferred into 4-mm cuvettes and electroporated. The parameters were 650 V, 25 μF and 99 Ohm for pulse 1 and 220 V, 1050 μF and 201 Ohm for pulse 2. Immediately afterwards, the cells were resuspended in medium with 10% CS FCS and seeded on 24-well plates. Hormones and/or AR antagonists were supplemented 24 h after electroporation in the presence or absence of serum and the cells were harvested after incubation for another 24 h. CAT assay was performed in cell extracts as described previously (Culig et al, 1994). The results of reporter gene assays were normalized according to protein levels and transfection efficacy which was determined by the Bio Orbit luciferase assay. The luciferase assay was performed according to the manufacturer's instructions. To test whether serum has any effect on the expression of the luciferase control plasmid, both LNCaP sublines were transfected with the $pGL_3$-control plasmid and treated with 10% CS FCS for 24 h. Afterwards, luciferase activity was determined. To confirm that reporter gene activity measured in CAT assays accurately reflects functional activity of the AR, LNCaP and LNCaP-abl cells were transfected with a control reporter gene plasmid lacking the androgen-response elements (TATA-CAT) and incubated in the absence or presence of androgen before CAT assay was performed.

### Determination of prostate-specific antigen mRNA levels by semiquantitative RT-PCR

LNCaP and LNCaP-abl cells were cultured in medium supplemented with 5% CS FCS in the absence or presence of androgen for 24 h. RNA was extracted according to the protocol described previously (Culig et al, 1993*b*). A PSA cDNA fragment and a cDNA fragment of the housekeeping gene β2-microglobulin were amplified in a duplex PCR. Amplification was started with PSA primers alone for 3 cycles. Thereafter, β2-microglobulin primers were added and the amplification was continued for another 20 cycles. The following PCR programme was used: a precycle of 15 min at 95°C followed by 23 cycles of 50 s at 94°C, 1 min at 56°C, 30 s at 72°C and a postcycle of 2 min at 73°C. The primers used for PSA detection were PSA 418/21 sense, 5′-GGC AGG TGC TTG TAG CCT CTC-3′ (fluorescence-labelled); and 939/21 antisense, CAC CCG AGC AGG TGC TTT TGC-3′. The primers used for the amplification of β2-microglobulin were sense, 5′-ATG CCT GCC GTG TGA ACC ATG T-3′; and antisense, 5′-AGA GCT ACC TGT GGA GCA ACC T-3′ (fluorescence-labelled). The fragments were separated by capillary gel electrophoresis on an ABI 310 Genetic Analyser (ABI, Foster City, CA, USA) and the fluorescence intensities of the fragments were measured using the ABI Prism Genescan Software. A PSA/β2-microglobulin ratio was calculated.

### Sequence analysis of AR cDNA fragments

Isolation of RNA from LNCaP and LNCaP-abl cells, cDNA synthesis, amplification of AR cDNA fragments and sequencing were performed as described previously (Culig et al, 1993*b*).

### Cytogenetic analysis

Metaphase spreads were prepared from subconfluent cell monolayers after 1 or 2 days in culture. The cells in log phase were microscopically checked for good mitotic index and harvested by colcemid and trypsin treatment using standard methods. Metaphase chromosomes were routinely analysed by trypsin-Giemsa (GTG) and quinacrine (QFQ) banding. GTG- and QFQ-banded chromosomes were recorded, karyotyped and processed by MacKtype 5.1 software on a Polyvar 2 microscope.

### CGH analysis

For CGH, metaphase chromosome preparation, DNA isolation and labelling, hybridization and signal detection were performed essentially as described previously (Kallioniemi et al, 1994). Briefly, test DNA was isolated from cultured LNCaP and LNCaP-abl cells and

 © 1999 Cancer Research Campaign

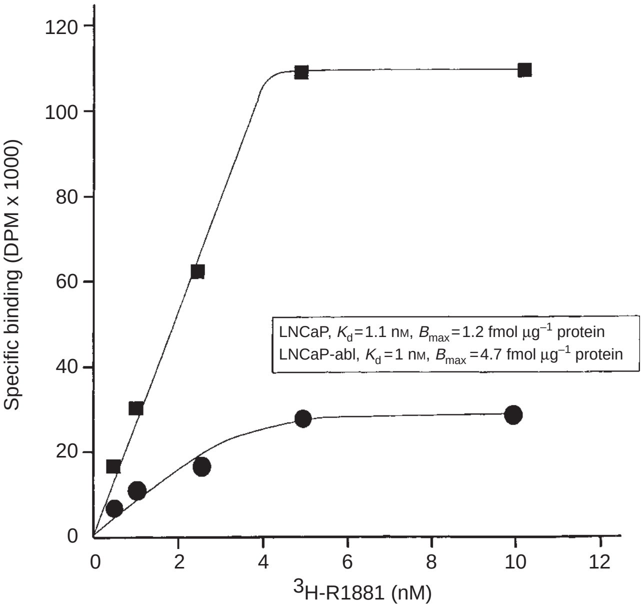

**Figure 2** Specific androgen binding in LNCaP and LNCaP-abl cells (passage 66). LNCaP cells were incubated in 75-cm$^2$ flasks for 48 h. Then the cells were harvested and incubated with increasing concentrations of $^3$H-R1881 at room temperature for 90 min. Non-specific binding was measured in the presence of a 200-fold molar excess of unlabelled R1881. After incubation the cells were washed twice with ice-cold medium and specifically bound radioactivity was determined

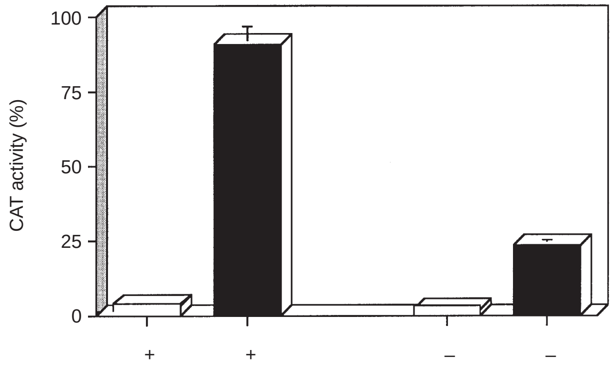

**Figure 3** Assessment of AR transcriptional activity in LNCaP (white bar) and LNCaP-abl (black bar) cells in the absence of androgen. The cells were cotransfected with the androgen-responsive reporter plasmid ARE$_2$TATA-CAT and the plasmid pGL$_3$-control containing a luciferase gene driven by the SV-40 promoter. The basal CAT activity in LNCaP and LNCaP-abl cells was measured in the presence or absence of serum. All CAT activities are expressed in relation to the CAT activity induced by 1 nM of R1881 in LNCaP cells (=100%, not shown); bars, s.d.

control DNA was prepared from blood of a healthy male. DNAs were labelled with biotin-16-dUTP and digoxigenin-11-dUTP, respectively, following standard nick translation protocols (Langer et al, 1981). A 1:1 probe mixture (400–600 ng) was hybridized in combination with unlabelled human Cot-1 DNA to normal male metaphase spreads. Biotin- and digoxigenin-labelled probes were visualized via fluorescein isothiocyanate (FITC) and tetrarhodamine isothiocyanate (TRITC) respectively. Chromosomes were identified on the basis of inverted grey level diamino-2-phenolidol (DAPI) banding. CGH results were evaluated using a digital image analysis system. Three single-colour images were recorded and processed by the MacProbe 3.3 software on the Leitz Aristoplan epifluorescence microscope. The microscope was equipped with a cooled integrating black and white CCD camera and a triple band-pass filter set in combination with single band-pass excitation filters to sequentially excite DAPI, FITC and TRITC without any registration shift between images. Green to red (tumour cell line to normal) fluorescence intensity ratios along the length of all chromosomes in the metaphase spreads were calculated, allowing an objective assignment of DNA sequence copy number changes of the tumour cell lines to chromosomal regions. After normalization of absolute fluorescence intensities within each metaphase to an average of 1.0, the results were plotted as green to red ratio profiles for each human chromosome from pter to qter. Typically, 3–5 high quality metaphases were analysed from each slide.

### Assessment of growth of LNCaP cells in vivo

Six-week-old male athymic nude mice (NMRI-strain, Bommice, Bomholdtgard, Denmark) were used in the in vivo experiments. The animals were housed in a pathogen-free facility. Animal treatment was according to *Guidelines for the Welfare of Animals in Experimental Neoplasia*. Mice that should be transplanted with LNCaP cells were supplemented with slow-release testosterone pellets (12.5 mg, 90-day release; Innovative Research of America, Sarasota, FL, USA). Prior to transplantation, LNCaP and LNCaP-abl cells were expanded in vitro. Exponentially growing cells were harvested and the cell pellet was resuspended in an appropriate volume of RPMI-1640 medium. The cell suspension was mixed 1:1 with matrigel to inject $1.5 \times 10^6$ cells in 0.1 ml subcutaneously (s.c.) into the left flank of each nude mouse. After the tumours had reached a size of 25–35 mm$^2$, either surgical castration under anaesthesia and testosterone pellet removal were performed or treatment with the anti-androgen bicalutamide (50 mg kg$^{-1}$ s.c. in benzyl benzoate/castor oil, 3 × weekly, synthesized at Schering AG) was started. Tumour size was measured weekly using a caliper (length × width of a tumour area in mm$^2$). All tumours were of a very similar shape. At the end of each experiment, tumours, prostate and seminal vesicles were dissected and weighed.

### RESULTS

### Establishment of an androgen-hypersensitive LNCaP subline

LNCaP cells obtained from the American Type Culture Collection were grown for 10 months in medium supplemented with 10% CS FCS. After 41 passages, the LNCaP-abl subline was established. Morphology of these cells was different to that of parental cells. Clusters of cells were observed in the subline generated after androgen ablation rather than uniform monolayers.

### Effects of long-term androgen ablation on proliferation of LNCaP cells

The basal proliferation rate of both LNCaP sublines was evaluated by determination of cell number after 3 days of growth. In the first series of experiments, LNCaP cells were grown in medium supplemented with FCS and LNCaP-abl cells in medium containing CS FCS. Until LNCaP-abl cells reached passage 61, their number was

*© 1999 Cancer Research Campaign* 

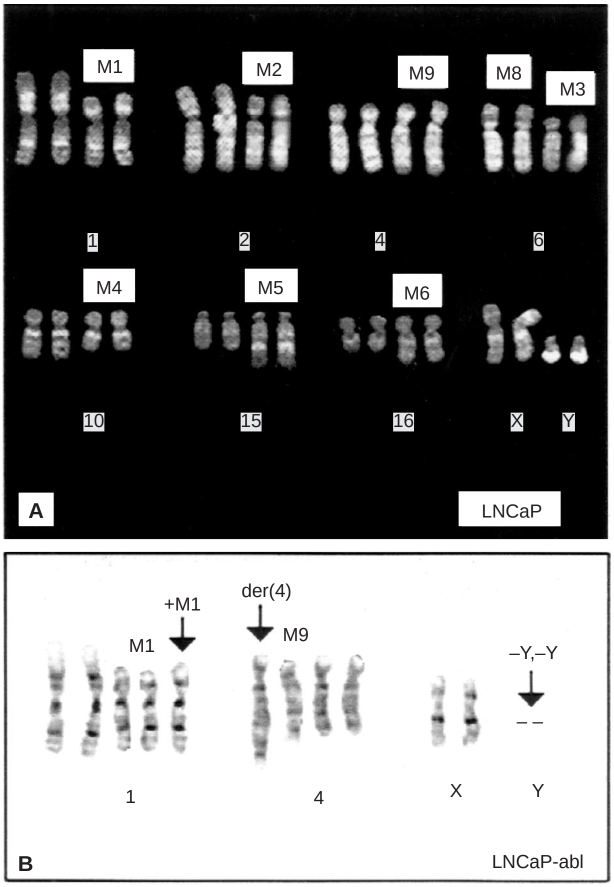

**Figure 4** Cytogenetic changes of the LNCaP prostate cancer cell line during in vitro propagation under steroid-free conditions. (**A**) Representative QFQ-banded partial karyotype of the parental LNCaP cell line illustrating the basic marker chromosomes M1 to M9. (**B**) Representative GTG-banded partial karyotype of the LNCaP-abl subline (passage 75). Chromosomal differences between karyotypes from LNCaP and LNCaP-abl were duplication of the marker M1, formation of a new der(4) marker derived from M8 and loss of the two Y-chromosomes (arrows)

15–20% lower than that of LNCaP cells. Beginning with passage 61, the basal proliferation rate of LNCaP-abl cells was quite similar to that of LNCaP cells. When LNCaP-abl cells were grown in FCS, their number was more than 2 times lower than the number of LNCaP cells. Finally, experiments in which both sublines were cultured in CS FCS revealed that LNCaP cells grow up to 2 times slower than LNCaP-abl cells in this medium. The effects of increasing concentrations of androgen and the non-steroidal anti-androgens hydroxyflutamide and bicalutamide on cellular proliferation were assessed by the MTT assay. The synthetic androgen R1881 was added at concentrations ranging from 0.001 to 1 nM. R1881 was used instead of DHT to minimize metabolic degradation of physiological androgen during incubation. Both the parental cell line and LNCaP-abl cells showed a biphasic response after incubation with androgen (Figure 1A). Maximal stimulation of the parental cell line was observed at 0.01 nM of R1881, whereas a tenfold lower concentration evoked maximal proliferative response in androgen-ablated cells. LNCaP-abl cells at passage 75 and later were inhibited by R1881 (Figure 1A). Hydroxyflutamide stimulated proliferation of both LNCaP and LNCaP-abl cells in a similar manner (data not shown). Surprisingly, bicalutamide, at concentrations between 100 nM and 10 μM, stimulated proliferation of

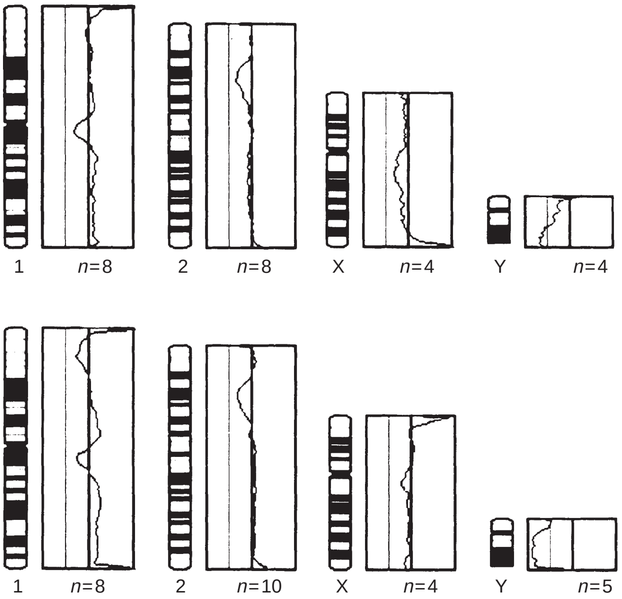

**Figure 5** CGH analysis in the parental LNCaP cell line (top) and in the LNCaP-abl subline (passage 70) demonstrating genetic changes affecting chromosomes 1, 2, X and Y (bottom). Average green to red fluorescence intensity ratio profiles from 4 to 10 analysed chromosomes are shown from pter to qter. Vertical lines represent green to red ratio values of 0, 0.5, 1, 1.5 and 2 (cut-off limits of losses and gains were taken at 0.75 and 1.25). The mean ratio values in LNCaP cells indicate a loss of the 2p13-p21 region as the only change whereas passage 70 of LNCaP-abl cells shows losses of 1p34-p36, 2p13-p21, Y and a gain of 1p22-qter. Idiograms are provided for approximate visual reference of the breakpoints. Heterochromatic chromosomal regions were excluded from the analysis

LNCaP-abl cells (Figure 1B). Positive effects of bicalutamide on growth of steroid-deprived LNCaP cells were observed in passages in which very low concentrations of R1881 stimulated proliferation as well as in those in which androgen inhibited cell growth.

### AR expression during propagation in steroid-depleted medium

AR protein level was estimated by ligand-binding assay with radioactively labelled R1881. Maximum binding was determined by means of Scatchard analysis. The number of binding sites in LNCaP cells was comparable with that previously reported by Kirschenbaum et al (1993) (1.2 vs 1.4 fmol $\mu g^{-1}$ protein). Our experiments revealed a gradual increase of AR during androgen ablation. The maximal fourfold increase in androgen binding was measured beginning with passage 66 (Figure 2) and remained essentially unchanged until passage 87. There was no change in AR binding affinity after long-term androgen deprivation. Interestingly, relative binding affinity of AR for bicalutamide was the same in LNCaP and LNCaP-abl cells (0.6). To investigate whether AR up-regulation also occurs after short-term androgen ablation, we cultured parental cells in medium with CS FCS for 4 days. Increased androgen binding was not measured in LNCaP cells after short-term androgen withdrawal.

### AR transcriptional activity

AR transcriptional activity was analysed by means of reporter gene transactivation assays. Reporter gene activity was assayed in

 *© 1999 Cancer Research Campaign*

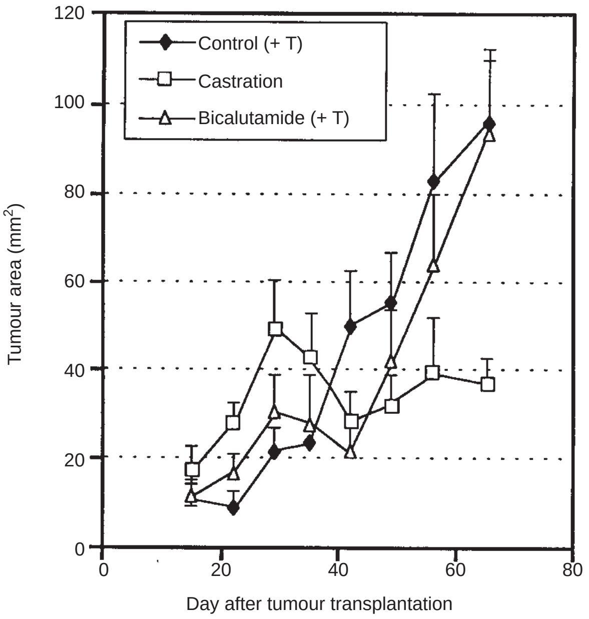

**Figure 6** Growth characteristics of the LNCaP tumour in male nude mice. LNCaP cells were transplanted s.c. into testosterone (T)-supplemented mice. Two weeks later, nude mice bearing tumours were randomized and divided into three groups (7–9 mice per group). The first group received no additional treatment (control) and in the second group mice were castrated and testosterone pellets were removed. Mice in the third group were treated with bicalutamide (50 mg $kg^{-1}$, 3 × weekly). The experiment was performed twice and similar results were obtained

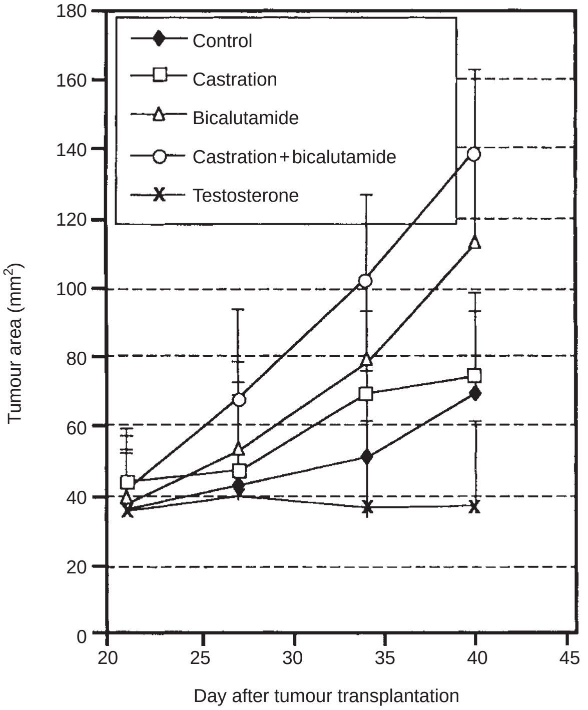

**Figure 7** Growth characteristics of the LNCaP-abl tumour in male nude mice. LNCaP-abl cells were transplanted s.c. into male nude mice. Two weeks later, nude mice bearing tumours were randomized and divided into five groups (7–10 mice per group). Treatment included castration, bicalutamide (50 mg $kg^{-1}$, 3 × weekly), castration plus bicalutamide (50 mg $kg^{-1}$, 3 × weekly) or testosterone supplementation. The experiment was performed twice and similar results were obtained extracts of LNCaP and LNCaP-abl cells which were transfected with the $ARE_2$ TATA-CAT construct and incubated with or without R1881 for 24 h. Two types of experiments were performed. In the first series of experiments, 5% CS FCS was present during incubation. In these experiments, parental LNCaP cells showed a very low reporter gene activity in the absence of androgen. In contrast, the basal CAT activity in androgen-ablated cells was approximately 30-fold higher in LNCaP-abl passages 63–66 than in control cells (Figure 3). This increased basal CAT activity was also measured up to passage 87 of LNCaP-abl cells. There was no elevation of basal reporter gene activity in LNCaP cells which were depleted of steroids for 4 days. Differences between control cells and the LNCaP-abl subline were also notable after androgenic stimulation. Reporter gene activity was induced by tenfold lower concentrations of androgen in LNCaP-abl than in LNCaP cells (Table 1). In the second series of experiments, reporter gene activity was measured in extracts of cells which were incubated in the absence of androgen and serum (Figure 3). The basal CAT activity was tenfold higher in LNCaP-abl than in LNCaP cells even under these experimental conditions. CAT reporter gene activities were normalized to luciferase activity of the pGL-3 control plasmid. In separate control experiments it was ensured that the expression of the control luciferase gene is equal in medium supplemented with FCS or CS FCS as well as in serum-free medium. In experiments in which the reporter plasmid TATA-CAT lacking the two androgen response elements was electroporated, CAT activity was similar in LNCaP and in LNCaP-abl cells. As expected, R1881 did not cause any stimulation of reporter gene activity in cells that expressed this vector.

**Table 1** Induction of CAT activity in LNCaP and LNCaP-abl cells transfected with the androgen-inducible reporter plasmid $ARE_2$TATA-CAT

| | **Cell line (fold induction of CAT activity over basal level ± s.d.)** | |
|---|---|---|
| **Compound** | **LNCaP** | **LNCaP-abl** |
| R1881 (0.01 nM) | 1.2 ± 0.1 | 3.8 ± 0.2 |
| R1881 (0.1 nM) | 3.5 ± 0.5 | 14.4 ± 1.1 |
| R1881 (1 nM) | 33.2 ± 2.2 | 42 ± 1.5 |
| OHF (100 nM) | 2.5 ± 0.1 | 6.3 ± 0.8 |
| OHF (1 μM) | 3.8 ± 0.5 | 9.1 ± 1.2 |
| OHF (10 μM) | 4.5 ± 0.3 | 18.3 ± 2.2 |
| R1881 (1 nM) + OHF (1 μM) | 31.6 ± 2.2 | 35 ± 2.8 |
| Bic (100 nM) | 0.9 ± 0.1 | 1.8 ± 0.2 |
| Bic (1 μM) | 0.9 ± 0.2 | 2.2 ± 0.2 |
| Bic (10 μM) | 1.1 ± 0.1 | 2.8 ± 0.4 |
| R1881 (1 nM) + Bic (1 μM) | 3.2 ± 0.6 | 39.2 ± 5.2 |

OHF = hydroxyflutamide; Bic = bicalutamide.

Next, we investigated the effects of the non-steroidal anti-androgens hydroxyflutamide and bicalutamide on AR-mediated reporter gene activity in androgen-ablated LNCaP cells. Agonistic effects of hydroxyflutamide in parental LNCaP cells were observed previously (Veldscholte et al, 1990, 1992). Stimulation of AR activity by hydroxyflutamide was 2.4- to 4-fold higher in LNCaP-abl cells than in LNCaP cells, thus confirming that these cells adapted to an androgen-depleted environment by generating a hyper-reactive AR (Table 1). Most interestingly, bicalutamide, a

© 1999 Cancer Research Campaign

**Table 2** Loss of the Y-chromosome evaluated by epifluorescence microscopy of QFQ-stained metaphases

| Cell line (passage) | Cells with XXYY (%) | Cells with XXY,-Y (%) | Cells with XX,-Y,-Y (%) | Total cells |
|---|---|---|---|---|
| LNCaP | 27 (90) | 3 (10) | 0 (0) | 30 |
| LNCaP-abl (75) | 0 (0) | 21 (70) | 9 (30) | 30 |
| LNCaP-abl (76) | 1 (7.1) | 8 (57.1) | 5 (35.7) | 14 |

pure AR antagonist in parental cells, displayed positive effects on reporter gene activity in LNCaP-abl cells. This compound inhibited CAT activity induced by R1881 in LNCaP cells. In contrast, the ability of bicalutamide to antagonize the effects of androgen on reporter gene activity was greatly impaired in LNCaP-abl cells generated after long-term androgen ablation (Table 1). This pattern of AR activation was also observed in later passages of LNCaP-abl cells (passages 67–87).

### PSA mRNA expression

In order to investigate how long-term androgen ablation affects the androgen-regulated PSA gene, LNCaP and LNCaP-abl cells were treated with R1881 for 24 h and PSA mRNA was determined by semiquantitative RT-PCR. Neither basal nor androgen-induced PSA mRNA levels differed significantly between androgen-ablated cells and controls. The mean PSA/β2-microglobulin ratio was 0.2 in untreated LNCaP and 0.3 in LNCaP-abl cells. In LNCaP cells treated with 1 nM of R1881 the mean PSA/β2-microglobulin ratio was 1.7 compared with LNCaP-abl cells that had a mean ratio of 1.3.

### AR sequence and cytogenetic analysis

Sequence analysis of AR cDNA fragments revealed that the AR in LNCaP-abl cells has the same sequence as the AR in LNCaP cells (threonine at codon 877 is replaced with alanine) (Veldscholte et al, 1990). The original cell line LNCaP and the LNCaP-abl subline were analysed by both conventional karyotyping and CGH. Figure 4 summarizes chromosome findings of the two cell lines. Both the parental cells and the LNCaP-abl subline had the same basic karyotype. This was essentially a near tetraploid karyotype with the consistent diploid markers M1 to M9 as reported by Gibas et al (1984) and Konig et al (1989). Three additional chromosomal changes compared to this basic karyotype were found in the LNCaP-abl subline: (i) an additional copy of marker M1 was present in LNCaP-abl cells; (ii) a new marker chromosome was formed from one copy of M8 (4q+); and (iii) a partial or complete loss of the Y-chromosome was observed (see also Table 2). Other microscopical chromosome changes did not occur during in vitro propagation.

Additionally, we have used CGH to identify clonal genetic changes arising from in vitro selection of the LNCaP-abl subline. CGH results from the two cell lines are shown in Figure 5. The parental LNCaP cell line showed loss of the region 2p13–p21. This change was also seen in the LNCaP-abl subline, whereas the gain of 1p22.3–qter and the loss of the Y chromosome were found only in the LNCaP-abl subline confirming in part conventional cytogenetic findings. Moreover, loss of 1p34–p36 in LNCaP-abl cells was found exclusively by CGH. None of passages of the LNCaP-abl subline tested showed amplification sites, in particular no amplification of the chromosomal region for the AR gene at Xq11–13.

**Table 3** Prostate and seminal vesicle weight in tumour-bearing male nude mice after androgen ablation, testosterone and anti-androgen treatment respectively

| | Seminal vesicle (relative weight in mg/100 g body weight) | s.d. | Prostate | s.d. |
|---|---|---|---|---|
| LNCaP | | | | |
| Control (+testosterone) | 821 | 203 | 190 | 32 |
| Castration | 101 | 11 | 48 | 14 |
| Bicalutamide (+testosterone) | 217 | 49 | 45 | 8 |
| LNCaP-abl | | | | |
| Control | 584 | 129 | 149 | 21 |
| Castration | 161 | 39 | 36 | 7 |
| Bicalutamide | 247 | 33 | 90 | 20 |
| Castration + bicalutamide | 100 | 24 | 30 | 3 |
| Testosterone | 964 | 151 | 227 | 66 |

### Growth of LNCaP sublines in nude mice

LNCaP cells were found to be tumorigenic in nude mice. Tumour growth rate was improved when LNCaP cells were injected in matrigel suspension and mice were supplemented with testosterone. A tumour take rate between 70 and 90% was achieved. In contrast, we found no tumour growth of LNCaP-abl cells (passages 73–87) in mice bearing testosterone pellets. However, LNCaP-abl cells exerted tumour growth in nude mice without androgen supplementation. Both cell lines showed similar growth rates and 2 weeks after transplantation palpable tumours were observed. After 3–4 weeks, a tumour size between 25 and 35 $mm^2$ was reached.

### Effects of anti-androgen therapy on in vivo growth of LNCaP and LNCaP-abl tumours

Treatment with bicalutamide (50 mg $kg^{-1}$, s.c.) resulted in an initial inhibition of LNCaP tumour growth which was followed by a relapse phase in which the tumours grew fast and reached the same size as controls. Androgen ablation by castration was effective in growth inhibition of the LNCaP xenotransplants and led to stabilization of tumour growth (Figure 6) for the indicated time period. This experiment was finished on day 65 when prostate and seminal vesicle weights were determined. These parameters served for assessment of the effect of treatment on non-malignant androgen-sensitive tissues (Table 3). In a similar experiment, we found a tumour relapse in castrated mice 70 days after tumour transplantation (data not shown). The LNCaP-abl xenografts grew well in intact male mice without androgen supplementation (Figure 7).

 © 1999 Cancer Research Campaign

Bicalutamide (50 mg kg$^{-1}$, s.c.) additionally stimulated tumour growth. In castrated mice, tumours grew equally well as in intact mice. However, tumours growing in castrated mice were even more sensitive to the stimulatory effects of bicalutamide. In contrast to original LNCaP cells, the implantation of testosterone pellets in the LNCaP-abl tumour-bearing mice resulted in stabilization of tumour size. As shown in Table 3, in both experiments bicalutamide reduced prostate and seminal vesicle weight, demonstrating the anti-androgenic effects of this drug on the proliferation of non-malignant hormone-dependent tissues.

### DISCUSSION

The major findings of this study are that after long-term androgen ablation the AR becomes active in the absence of hormones and serum and the pure AR blocker bicalutamide acquires agonistic properties. This agonistic activity of bicalutamide was demonstrated in cotransfection-transactivation assays and in experiments in which tumour cell growth in vitro and in vivo was assessed. High AR activity in the absence of measurable androgen in LNCaP-abl cells suggested the development of a hypersensitive AR during propagation in steroid-depleted medium. One possible interpretation of our data is that very low levels of steroid hormones which may be still present in CS FCS stimulate AR activity in LNCaP-abl cells. We did not detect any testosterone, oestradiol and progesterone in CS FCS with RIAs. However, we cannot completely rule out the possibility that concentrations of these hormones below assay detection limits are present in CS FCS. We hypothesized that non-steroidal substances present in serum are, at least in part, responsible for an increased basal activity of the AR. Recent studies have shown that growth factors, peptide hormones and substances which directly activate protein kinases participate in AR signalling. Ligand-independent activation of the AR by growth factors and protein kinase A activators and synergistic effects of these activators and androgen were reported (Culig et al, 1994, 1997*a*; Nazareth and Weigel, 1996). Furthermore, androgen-like effects of epidermal growth factor and keratinocyte growth factor during sexual development and AR-dependent stimulation of PSA secretion in explants of prostate tissue by a cyclic adenosine monophosphate analogue were described (Gupta et al, 1996; Nakhla et al, 1997; Thomson et al, 1997). We aimed to obtain more information on AR activation in LNCaP-abl cells and reduced the influence of serum in subsequent experiments as much as possible. Constitutive activity of the AR, although diminished, was noted even under stringent experimental conditions. The fact that in LNCaP-abl cells tenfold lower concentrations of R1881 than in original cells induced AR function supports the concept of the development of receptor hypersensitivity during androgen ablation in vitro. Experiments in which reporter gene activity was measured after treatment with anti-androgens provided additional evidence for AR hyperreactivity as an adaptation to androgen depletion. Agonistic effects of hydroxyflutamide in LNCaP cells were well-documented in the past (Veldscholte et al, 1990, 1992). Long-term androgen ablation apparently potentiates agonism of hydroxyflutamide. In contrast to hydroxyflutamide, the non-steroidal anti-androgen bicalutamide is an AR antagonist in parental LNCaP cells but acts as an agonist in LNCaP-abl cells. The induction of reporter gene activity was lower by bicalutamide than by hydroxyflutamide; however, the induction by bicalutamide was constantly observed at concentrations ranging between 100 nM and 10 μM. Although AR transcriptional activity in LNCaP-abl cells was dramatically increased, there was a paradoxical finding that the expression of the androgen-regulated PSA gene is unchanged. At present, there is no explanation for this unexpected observation. However, similar findings were reported for hypersensitive MCF-7 cells by Jeng et al (1998). Those cells also show an increased oestrogen receptor activity but no elevation of the oestrogen-regulated progesterone receptor and transforming growth factor-α genes. Taken together, our results and those of Jeng et al suggest that not all steroid-regulated genes are up-regulated after long-term steroid depletion in tumour cells.

We speculated that either an alteration in the AR sequence or AR gene amplification may be an underlying mechanism responsible for the dramatic changes in the transcriptional activity of the LNCaP-abl AR. However, the sequence of the AR in the LNCaP-abl subline does not differ from the sequence of the AR in original cells. Amplification of the AR gene was demonstrated in about 30% of advanced prostate tumours (Visakorpi et al, 1995; Cher et al, 1996; Koivisto et al, 1997). Our experiments revealed that this mechanism is not operative in LNCaP-abl cells. Further studies on elucidation of molecular mechanisms responsible for alterations in AR function will focus on possible changes in interaction of the AR with receptor-associated coactivator and co-repressor proteins and on alterations in AR phosphorylation. Several nuclear receptor coactivators were found to enhance AR-mediated transactivation (Voegel et al, 1996; Yeh and Chang, 1996; Ikonen et al, 1997; Froesch et al, 1998). Miyamoto and colleagues reported that anti-androgens can promote the interaction between the AR and its coactivator ARA70, thus enhancing AR transcriptional activity (Miyamoto et al, 1997). The hypothesis that changes in the expression and function of these co-regulatory proteins occur during propagation in androgen-depleted environment warrants further investigation.

The AR is expressed in early stage prostate cancer, in relapsed tumours and in metastatic lesions from patients who failed androgen ablation therapy (van der Kwast et al, 1991; Hobisch et al, 1995, 1996; Taplin et al, 1995). Mutant ARs were discovered in some primary tumours and, in higher percentage, in metastases (reviewed by Culig et al, 1997b). These receptors are frequently promiscuous responding to non-androgenic steroids and AR antagonists and, most probably, play a role in prostate tumour progression. Changes in AR activity described in the present report, if not restricted to LNCaP cells, offer another possibility for prostate tumour cells to escape ablation therapy. This fact may additionally complicate the design of an appropriate endocrine treatment for metastatic cancer of the prostate.

The cells generated in our laboratories and those characterized by Kokontis et al (1994) share some common features. The biphasic proliferative response shifted to lower androgen concentrations in LNCaP-abl, LNCaP-I (developed by Kokontis and associates after 20–30 passages in androgen-depleted medium) and in LNCaP-R cells (characterized after another 20–30 passages). AR mRNA and protein increased during progression in cell culture in LNCaP-I and LNCaP-R cells (Kokontis et al, 1994). Kokontis and associates also reported on changes in AR activity in steroid-depleted cells. Their observation that the induction of CAT activity by R1881 is about sevenfold more efficient in androgen-ablated than in original cells complies with our data. Finally, growth of LNCaP-R cells in nude mice was inhibited by DHT, whereas finasteride, an inhibitor of the enzyme 5α-reductase, showed stimulation of tumour growth (Umekita et al, 1996). While this manuscript was in preparation, Kokontis et al (1998) reported that repression of long-term androgen-ablated cells in vitro is associated with the accumulation of the cyclin-dependent kinase inhibitor $p27^{Kip1}$

© 1999 Cancer Research Campaign

(1998). Although the LNCaP subline developed in our laboratories and those described by Kokontis and associates (LNCaP-R1 and -R2) are both repressed by androgen, they differ in their responsiveness to bicalutamide. This compound did not show a stimulatory effect on LNCaP-R proliferation (Kokontis et al, 1998). Hence, phenotypical diversities after long-term androgen ablation may occur. The anti-androgen bicalutamide was not tested in LNCaP in vivo models so far. In our study, LNCaP tumours regressed after castration, whereas bicalutamide treatment led to a transient retardation of growth of LNCaP tumours in mice followed by relapse. The size of prostate glands and seminal vesicles in bicalutamide-treated animals was reduced comparably to that in castrated mice demonstrating a strong anti-androgenic effects of this compound. Since bicalutamide stimulated proliferation of the LNCaP-abl subline in cell culture, we questioned whether this anti-androgen can also promote its growth in nude mice. We demonstrated that bicalutamide strongly enhances growth of the LNCaP-abl tumour in mice, whereas testosterone inhibits its proliferation. Our findings clearly point out that long-term androgen ablation favours selection of tumour cells in which anti-androgens have agonistic activity thus underscoring the need to develop new anti-androgens without partial agonistic activity.

Cytogenetic analysis of the parental human prostate cancer cell line LNCaP used in this study revealed essentially the karyotype of the cell line originally developed by Horoszewicz et al (1983) and chromosomally characterized by Gibas et al (1984) and Konig et al (1989). Thus, the basic karyotype of the original cell line was well preserved. Duplication of the marker M1, interpreted as a der(1) t(1;15) (p22.3; q24), was found also in other LNCaP sublines that were established during in vitro propagation (Konig et al, 1989). Data from those cell lines suggest that duplication of this marker is rather a result of cytogenetic evolution during long-term in vitro culture than a specific response of LNCaP cells to androgen-free conditions. CGH analyses of the LNCaP cell line and androgen-independent sublines derived after in vivo passages through athymic mice were recently performed by Hyytinen et al (1997). In contrast to the loss of the 2p13–p21 region which was the only change in copy number in our study, these authors detected different changes such as losses of chromosomal regions 1p34–p36, 2, 6cen–q23, 13q and gains at 3q13–qter and 19p in the parental LNCaP cell line. This might be the consequence of the use of different cell material since the parental LNCaP cell line used by these authors was not obtained from ATCC. It is remarkable that the loss of 1p34–p36 was also found in our study, however, not in the parental LNCaP cell line but in the androgen hypersensitive LNCaP-abl subline. Notably, none of the consistent genetic changes which Hyytinen and associates found in the in vivo selected sublines were detected in our in vitro selected LNCaP-abl subline.

In summary, our results show for the first time that the anti-androgen bicalutamide acquires agonistic properties during long-term androgen ablation and that this phenomenon may be relevant to prostate cancer progression. The findings reported in the present study may have repercussions on the natural course of prostate cancer with androgen deprivation and on strategies of therapeutic intervention.

### ACKNOWLEDGEMENTS

This work was supported by grant SFB 002 F203 of the Austrian Science Foundation (FWF). The authors thank Prof. G Daxenbichler and Drs MV Cronauer and C Nessler for helpful discussions, G Sierek, G Hölzl, U Plawenn, M Pöschl, E Tafatsch, P Dertschnig, A Goede and M Sommer for excellent technical assistance, and M Trebo, M Neuhauser and R Schober for editorial assistance. We thank Essex Pharma (Munich, Germany) for providing hydroxyflutamide for this study.

### REFERENCES

Cher ML, Bova GS, Moore DH, Small EJ, Carroll PR, Pin SS, Epstein JI, Isaacs WB and Jensen RH (1996) Genetic alterations in untreated metastases and androgen-independent prostate cancer detected by comparative genomic hybridization and allelotyping. *Cancer Res* **56**: 3091–3102

Culig Z, Hobisch A, Cronauer MV, Cato ACB, Hittmair A, Radmayr C, Eberle J, Bartsch G and Klocker H (1993*a*) Mutant androgen receptor detected in an advanced-stage prostatic carcinoma is activated by adrenal androgens and progesterone. *Mol Endocrinol* **7**: 1541–1550

Culig Z, Klocker H, Eberle J, Kaspar F, Hobisch A, Cronauer MV and Bartsch G (1993*b*) DNA sequence of the androgen receptor in prostatic tumor cell lines and tissue specimens assessed by means of the polymerase chain reaction. *Prostate* **22**: 11–22

Culig Z, Hobisch A, Cronauer MV, Radmayr C, Trapman J, Hittmair A, Bartsch G and Klocker H (1994) Androgen receptor activation in prostatic tumor cell lines by insulin-like growth factor-I, keratinocyte growth factor, and epidermal growth factor. *Cancer Res* **54**: 5474–5478

Cronauer MV, Klocker H, Talasz H, Geisen FH, Hobisch A, Radmayr C, Böck G, Culig Z, Schirmer M, Reissigl A, Bartsch G and Konwalinka G (1996) Inhibitory effects of the nucleoside analogue gemcitabine on prostatic carcinoma cells. *Prostate* **28**: 172–181

Culig Z, Hobisch A, Hittmair A, Cronauer MV, Radmayr C, Zhang J, Bartsch G and Klocker H (1997*a*) Synergistic activation of androgen receptor by androgen and luteinizing hormone-releasing hormone in prostatic carcinoma cells. *Prostate* **32**: 106–114

Culig Z, Hobisch A, Hittmair A, Cronauer MV, Radmayr C, Bartsch G and Klocker H (1997*b*) Androgen receptor gene mutations in prostate cancer. Implications for disease progression and therapy. *Drugs Aging* **10**: 50–58

Dijkman GA and Debruyne FM (1996) Epidemiology of prostate cancer. *Eur Urol* **30**: 281–295

Froesch BA, Takayama S and Reed JC (1998) BAG-1L protein enhances androgen receptor function. *J Biol Chem* **273**: 11660–11666

Gibas Z, Becher R, Kawinski E, Horoszewicz J and Sandberg AA (1984) A high-resolution study of chromosome changes in a human prostate carcinoma cell line. *Cancer Genet Cytogenet* **11**: 399–404

Gupta C, Chandorkar A and Nguyen AP (1996) Activation of androgen receptor in epidermal growth factor modulation of fetal mouse sexual differentiation. *Mol Cell Endocrinol* **123**: 89–95

Hobisch A, Culig Z, Radmayr C, Bartsch G, Klocker H and Hittmair A (1995) Distant metastases from prostatic carcinoma express androgen receptor protein. *Cancer Res* **55**: 3068–3072

Hobisch A, Culig Z, Radmayr C, Bartsch G, Klocker H and Hittmair A (1996) Androgen receptor status of lymph node metastases from prostate cancer. *Prostate* **28**: 129–135

Horoszewicz JS, Leong SS, Kawinski E, Karr JP, Rosenthal H, Chu TM, Mirand EA and Murphy GP (1983) LNCaP model of human prostatic carcinoma. *Cancer Res* **43**: 1809–1818

Hyytinen ER, Thalmann GN, Zhau HE, Karhu R, Kallioniemi OP, Chung LWK and Visakorpi T (1997) Genetic changes associated with the acquisition of androgen-independent growth, tumorigenicity and metastatic potential in a prostate cancer model. *Br J Cancer* **75**: 190–195

Ikonen T, Palvimo JJ and Jänne OA (1997) Interaction between the amino- and carboxyl-terminal regions of the rat androgen receptor modulates transcriptional activity and is influenced by nuclear receptor coactivators. *J Biol Chem* **272**: 29821–29828

Jeng M-H, Shupnik MA, Bender TP, Westin EH, Bandyopadhyay D, Kumar R, Masamura RJ and Santen RJ (1998) Estrogen receptor expression and function in longterm estrogen-deprived human breast cancer cells. *Endocrinology* **139**: 4164–4174

Kaighn ME, Shankar Narayan K, Ohnuki Y, Lechner JF and Jones LW (1979) Establishment and characterization of a human prostatic carcinoma cell line (PC-3). *Invest Urol* **17**: 16–23

Kallioniemi OP, Kallioniemi A, Piper J, Isola J, Waldman FM, Gray JW and Pinkel D (1994) Optimizing comparative genomic hybridization for analysis of DNA

 *© 1999 Cancer Research Campaign*

sequence copy number changes in solid tumors. *Genes Chromosomes Cancer* **10**: 231–243
Kirschenbaum A, Ren M and Levine AC (1993) Enhanced androgen sensitivity in serum-free medium of a subline of the LNCaP human prostate cancer cell line. *Steroids* **58**: 439–444
Koivisto P, Kononen J, Palmberg C, Tammela T, Hyytinen E, Isola J, Trapman J, Cleutjens K, Noordzij A, Visakorpi T and Kallioniemi OP (1997) Androgen receptor gene amplification: a possible molecular mechanism for androgen deprivation therapy failure in prostate cancer. *Cancer Res* **57**: 314–319
Kokontis J, Takakura K, Hay N and Liao S (1994) Increased androgen receptor activity and altered c-myc expression in prostate cancer cells after long-term androgen deprivation. *Cancer Res* **54**: 1566–1573
Kokontis JM, Hay N and Liao S (1998) Progression of LNCaP prostate tumor cells during androgen deprivation: hormone-independent growth, repression of proliferation by androgen, and role for p27Kip 1 in androgen-induced cell cycle arrest. *Mol Endocrinol* **12**: 941–953
Konig JJ, Kamst E, Hagemeijer A, Romijn JC, Horoszewicz J and Schröder FH (1989) Cytogenetic characterization of several androgen responsive and unresponsive sublines of the human prostatic carcinoma cell line LNCaP. *Urol Res* **17**: 79–86
Langer PR, Waldrop AA, Ward DC (1981) Enzymatic synthesis of biotin-labeled polynucleotides: novel nucleic acid affinity probes. *Proc Natl Acad Sci USA* **78**: 6633–6637
Lee C, Sutkowski DM, Sensibar JA, Zelner D, Kim I, Amsel I, Shaw N, Prins GS and Kozlowski JM (1995) Regulation of proliferation and production of prostate-specific antigen in androgen-sensitive prostatic cancer cells, LNCaP, by dihydrotestosterone. *Endocrinology* **136**: 796–805
Miyamoto H, Yeh S, Wilding G and Chang C (1997) Promotion of agonist activity of antiandrogens by the androgen receptor coactivator, ARA70, in human prostate cancer DU145 cells. *Proc Natl Acad Sci USA* **95**: 7379–7384
Nakhla AM, Romas NA and Rosner W (1997) Estradiol activates the prostate androgen receptor and prostate-specific antigen secretion through the intermediacy of sex hormone-binding globulin. *J Biol Chem* **272**: 6838–6841
Nazareth LV and Weigel NL (1996) Activation of the human androgen receptor through a protein kinase A signaling pathway. *J Biol Chem* **271**: 19900–19907
Romijn JC, Verkoelen CF and Schroeder FH (1988) Application of the MTT assay to human prostate cancer cell lines in vitro: establishment of test conditions and assessment of hormone-stimulated growth and drug-induced cytostatic and cytotoxic effects. *Prostate* **12**: 99–110
Scher HI and Kolvenbag GJ (1997) The antiandrogen withdrawal syndrome in relapsed prostate cancer. *Eur Urol* **31**: 3–7
Schuurmans ALG, Bolt J, Voorhorst M, Blankenstein RA and Mulder E (1988) Regulation of growth and epidermal growth factor receptor levels of LNCaP prostatic tumor cells by different steroids. *Int J Cancer* **42**: 917–922
Stone KR, Mickey DD, Wunderli H, Mickey GH and Paulson DF (1978) Isolation of a human prostate carcinoma cell line (DU 145). *Int J Cancer* **21**: 274–281
Taplin ME, Bubley GJ, Shuster TD, Frantz ME, Spooner AE, Ogata GK, Keer HN and Balk SP (1995) Mutation of the androgen receptor gene in metastatic androgen-independent prostate cancer. *New Engl J Med* **332**: 1393–1398
Thomson AA, Foster BA and Cunha GR (1997) Analysis of growth factor and receptor mRNA levels during development of the rat seminal vesicle and prostate. *Development* **124**: 2431–2439
Tilley WD, Wilson CM, Marcelli M and McPhaul MJ (1990) Androgen receptor gene expression in human prostate carcinoma cell lines. *Cancer Res* **50**: 5382–5386
Umekita Y, Hiipakka RA, Kokontis JM and Liao S (1996) Human prostate tumor growth in athymic mice: inhibition by androgens and stimulation by finasteride. *Proc Natl Acad Sci USA* **93**: 15152–15157
Van der Kwast TH, Schalken J, Ruizeveld de Winter JA, van Vroonhoven CCJ, Mulder E, Boersma W and Trapman J (1991) Androgen receptors in endocrine-therapy-resistant human prostate cancer. *Int J Cancer* **48**: 189–193
Veldscholte J, Ris-Stalpers C, Kuiper GGJM, Jenster G, Berrevoets C, Claassen E, van Rooij HCJ, Trapman J and Brinkmann AO (1990) A mutation in the ligand binding domain of the androgen receptor of human LNCaP cells affects steroid binding characteristics and response to anti-androgens. *Biochem Biophys Res Commun* **17**: 534–540
Veldscholte J, Berrevoets CA, Zegers ND, van der Kwast TH, Grootgoed JA and Mulder E (1992) Hormone-induced dissociation of the androgen receptor-heat-shock protein complex: use of a new monoclonal antibody to distinguish transformed from nontransformed receptors. *Biochemistry* **31**: 7422–7430
Visakorpi T, Hyytinen E, Koivisto P, Tanner M, Keinänen R, Palmberg C, Palotie A, Tammela T, Isola J and Kallioniemi OP (1995) In vivo amplification of the androgen receptor gene and progression of human prostate cancer. *Nature Gen* **9**: 401–406
Voegel JJ, Heine MJ, Zechel C, Chambon P and Gronemeyer H (1996) TIF2, a 160 kDa transcriptional mediator for the ligand-dependent activation function AF-2 of nuclear receptors. *EMBO J* **15**: 3667–3675
Wilding G, Chen M and Gelmann EP (1989) Aberrant response in vitro of hormone-responsive prostate cancer cells to antiandrogens. *Prostate* **14**: 103–115
Yeh S and Chang C (1996) Cloning and characterization of a specific coactivator, ARA70, for the androgen receptor in human prostate. *Proc Natl Acad Sci USA* **93**: 5517–5521

*© 1999 Cancer Research Campaign*
